# Measurement of Urinary N-Telopeptides and Serum C-Telopeptides from Type I Collagen Using a Lateral Flow-Based Immunoassay

**DOI:** 10.3390/s130100165

**Published:** 2012-12-24

**Authors:** Kyoung Min Lee, Min Ho Lee, Chin Youb Chung, Woo Kyeong Seong, Sang Dae Lee, Moon Seok Park

**Affiliations:** 1 Department of Orthopaedic Surgery, Seoul National University Bundang Hospital, Kyungki 463-707, Korea; E-Mails: oasis100@empal.com (K.M.L.); pmsmed@gmail.com (M.S.P.); 2 Korea Electronics Technology Institute, Yatap-dong, Bundang-gu, Sungnam, Kyungki 463-816, Korea; E-Mails: mhlee@keti.re.kr (M.H.L.); seong@keti.re.kr (W.K.S.); 3 IM Electronics, Maetan-dong, Suwon, Kyungki 443-370, Korea; E-Mail: sdlee@im-electronics.com

**Keywords:** osteoporosis, urine N-telopeptide, serum C-telopeptide, lateral flow immunoassay, bone turnover marker

## Abstract

Measuring bone turnover markers could detect early stages of osteoporosis and early responses to anti-osteoporotic treatments. Currently, commonly used bone turnover markers, N-telopeptides (NTx) and C-telopeptides (CTx), are measured using ELISA tests, which demands time and increases cost. Bone turnover markers need to be measured more easily for general use. Lateral flow-based immunoassay would be an appropriate method for this context. This study was performed to investigate the precision of a newly developed lateral flow-based immunoassay for measuring the urinary NTx and serum CTx, and their correlations with ELISA measurements. Urine NTx and serum CTx concentrations were determined by photoscan of newly developed strips, using a lateral flow-based immunoassay for 36 subjects (mean age 66.2 years, SD 7.5 years; four males and 32 females). Repeated measurement of urinary NTx and serum CTx were performed three times, using this technology for a precision test. The correlation of the lateral flow-based immunoassay with the ELISA measurements was analyzed. Precision of the newly developed lateral flow based immunoassay was 0.974 (ICC, 95% confidence interval, 0.955 to 0.986) and 0.995 (ICC, 95% confidence interval, 0.991 to 0.997) for urinary NTx and serum CTx, respectively. The correlation of lateral flow based immunoassay with ELISA was 0.913 for urinary NTx and 0.872 for serum CTx. These results suggest that measuring the urinary NTx and serum CTx, using a lateral flow-based immunoassay, is a relevant method for point-of-care testing and screening of bone resorption markers.

## 1. Introduction

Osteoporosis is one of the most serious diseases of an aging population, causing osteoporotic fractures and raising mortality and morbidity [1–4]. Standard diagnosis usually depends on measuring the bone mineral density using a dual energy X-ray absorptiometry (DEXA) [5]. Although DEXA is a useful and direct tool for measuring the bone mineral density, it exposes subjects to radiation and does not provide information regarding bone formation or resorption, unless the change of bone mineral density is prominent.

Bone turnover markers are the metabolic products of bone resorption or materials that mediate bone metabolism. These markers can provide information regarding bone formation or resorption before the structural changes of bone occur [6–9]. Therefore, their clinical utility draws attention as an examination tool, following the treatment. Of these, urinary N-telopeptide (NTx) and serum C-telopeptide (CTx) appears to be the most promising bone resorption markers [10,11]. However, they are currently measured by ELISA methods, which are somewhat costly and slow. Considering the increasing incidence of osteoporosis in aging populations, bone turnover markers need to be measured more easily and economically, as well as accurately and precisely.

Lateral flow-based immunoassay represents a simple and well established technology to detect the presence of a target material by an immune reaction without the need for specialized and costly equipment. It is used in medical diagnostics for field use, point of care testing, or laboratory use [12]. This method is capable of providing high sensitivity, specificity, and good stability and easy integration with on board electronics [13].

This study was to investigate the clinical feasibility of measuring urinary NTx and serum CTx, using a lateral flow-based immunoassay, estimating precision and correlation with the ELISA method. Recognition of the analyte was done by binding to NTx- and CTx receptor conjugated to gold colloids, enabling the visualization.

## 2. Experimental Section

This prospective study was approved by the Institutional Review Board at our institution. Informed consent was obtained from all subjects. Urine and serum samples were collected between December 2009 and January 2010, from 36 consecutive subjects who consented to participate. Patients who underwent DEXA, as well as blood and urine tests for osteoporosis were included. Patients with renal dysfunction were excluded from the study.

### 2.1. Development of the Strip for Lateral Flow Based Immunoassay

The components used for this lateral flow immunoassay are the assembly membrane, conjugate pad, sample pad, wick, and backing materials, which were purchased from Millipore (Bedford, MA, USA). Nitrocellulose material as an analytical membrane with pore sizes of 15 micron was used. For the assay performance, the sample pad, which is made of glass fiber, was pretreated with proteins (hsCRP and PSA), surfactants (25% sucrose and 0.5% Tween 20), and polymers (nitrocellulose) prior to the immunoassays. The strips were dried for 1 hour at room temperature after receptor immobilization. In these experiments, urine and serum samples were used as analyte-containing samples. To provide rigidity and easy handling of the strip polystyrene material was used for the backing material to which all the components were laminated. NTx ELISA kit were purchased from Osteomark (Inverness Medical, Pleasanton, CA, USA), and CTx ELISA kit were purchased from MyBioSource (San Diego, CA, USA). Goat anti-rabbit IgG and rabbit IgG were obtained from Sigma (St. Louis, MO, USA).

### 2.2. Preparation NTx and CTx Conjugates

Colloidal gold (or gold nanoparticle, AuNP) was prepared by sodium citrate reduction, as previously described by Frens and Turkevivch *et al.* [14,15]. Briefly, 0.01% of tetrachloroauric acid trihydrate (HAuCl_4_·3H_2_O, Sigma Aldrich) was dissolved in 250 mL of boiling ultrapure water by vigorous stirring. Sodium citrate solution (15 mL, 1% solution) was then rapidly added. This caused the faintly blue solution to become dark red, which indicates the formation of monodispersed spherical particles. The gold nanoparticles formed were spherical and had a diameter of approximately 17 nm. Colloid suspensions were stored in a refrigerator for further use. Characterization of colloidal gold has been explained in our previous work [16].

**Figure 1.**
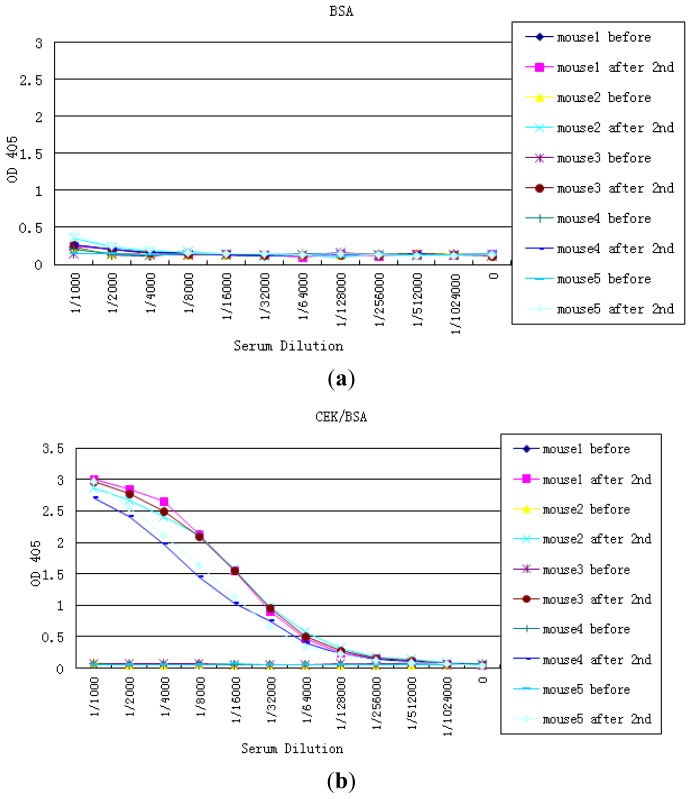
Antisera from the five Balb/C mice test using direct ELISA. (**a**) microplates coated with BSA and (**b**) microplates coated with the BSA-conjugated peptide.

For the production of antibodies against NTx and CTx, the following protocols were applied: briefly, custom peptides were systematically synthesized in the service lab (NTx: α1 (I)(Y)DEKSTGG(I)–α2 (I)QYDGKGVG(L) [17], CTx: H-CEKAHDGGR-OH) followed by peptide-carrier bioconjugation, immunization and antisera production. After immunization, the antisera from the five mice were tested by direct ELISA. The BSA-conjugated peptide was coated on microplates and serial dilutions of pre-immune and antisera were tested (Figure 1). Standard protocols of fusion with mouse myeloma cells were employed. Hybridoma supernatants were screened by ELISA using CEK-BSA conjugated peptides for selecting desirable monoclonal antibodies. Positive clones were subcloned, expanded and stored.

For the preparation of colloidal gold conjugates, we adjusted the pH value to 8.5 with 0.2 mol/L K_2_CO_3_ solution and added 100 mg/mL to the 1 mL colloidal gold solution. After 30 min incubation at room temperature, the colloidal gold conjugates were centrifuged two or three times at 30,000 rpm for 25 min to discard supernatants. The antibodies (100 μg/mL) were conjugated to colloidal gold and a conjugated pad with a size of 7.2 cm was saturated with 15 μL PBS solution with antibody conjugated colloidal gold.

### 2.3. Preparation of the Strip Assembly

**Figure 2.**
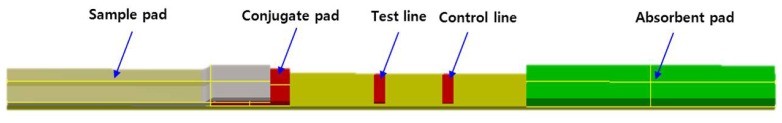
Schematic diagram of the sandwich lateral flow immunoassay strip.

The main body of the strip consisted of polystyrene backing, sample pad, conjugate pad, absorbent pad and nitrocellulose membrane. Anti-CTx and NTx antigen were immobilized on the nitrocellulose membrane (Figure 2, test line) by a dispenser system, and goat anti-mouse IgG antibodies were immobilized (Figure 2, control line) on the membrane. The assembled strip was dried overnight at 37 °C and stored until use. The NTx and CTx antibodies were immobilized at a test line and control line, respectively.

### 2.4. Precision of the Lateral Flow Based Immunoassay for Repetitively Measuring Urinary NTx and Serum CTx Levels

Urinary NTx levels and serum CTx levels were measured three times with an interval of two hours using the lateral flow-based immunoassay. The precision of the repeated measurements was determined using intraclass correlation coefficients (ICCs). Urine and serum samples were stored in a refrigerator at 4 °C to minimize the denaturation of the bone resorption markers (urinary NTx and serum CTx).

**Figure 3.**
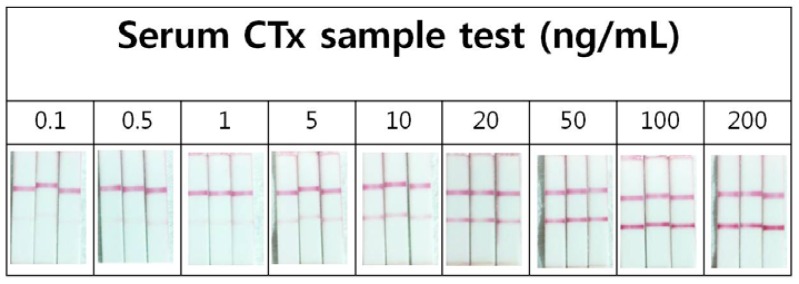
The predetermined concentrations of testing antigen were pretested three times before measuring patients' serum samples. Upper band is control line and lower band is test line.

**Figure 4.**
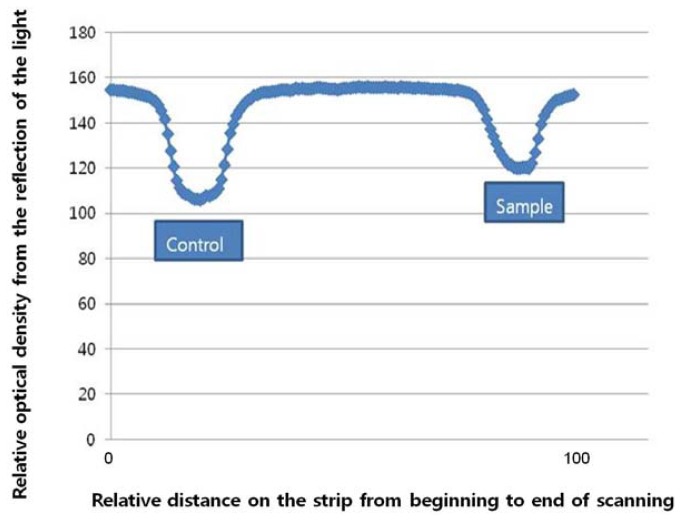
Strips measuring CTx 15 ng/mL was scanned by Image J and the concentration was calculated by comparing the control line and sample (test) line.

To obtain the quantitative results, the optical images of the strip were captured using a digital camera and their images were automatically converted into gray scale, using image J software. The test line quantifies the target and the control line confirms adequate conditions (Figure 2). The intensities of each test line signal were then analyzed for the quantification. This was performed with serum samples spiked with predetermined concentrations of target for the sensitivity test (Figures 3 and 4).

**Figure 5.**
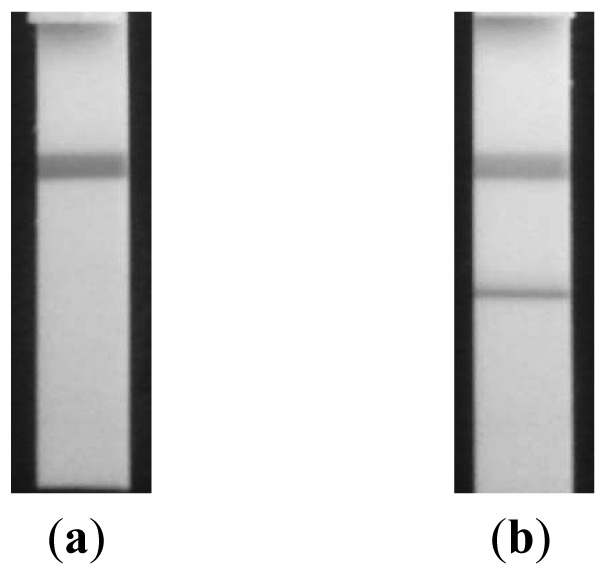
Selectivity test for the strips used. (**a**) normal serum and (**b**) serum spiked with target samples.

Figure 5 shows their specificity against target. In this experiment, serum and spiked serum were used for the measurements resulting that a clear line appeared only in spiked serum while no sign of reaction was observed in control (Figure 5).

### 2.5. Correlation of the Lateral Flow-Based Immunoassay for Measuring Urinary NTx and Serum CTx with ELISA Measurements

The correlation between the lateral flow-based immunoassay and ELISA were evaluated for urinary NTx and serum CTx, respectively. ELISA kits were utilized as gold standards of measuring urinary NTx and serum CTx. Osteomark (Osteomark; Ostex International, Princeton, NJ, USA) and Elecsys 2010 (Elecsys; Roche, Indianapolis, IN, USA) were used for urinary NTx and serum CTx, respectively. Measurements of urinary NTx, using ELISA, were normalized with respect to creatinine concentration, where measured optical densities were calculated as bone collagen equivalents (BCE), using a four-parameter calibration method [18]. Urinary creatinine (Cr) levels were measured using a creatinine colorimetric detection kit (Luminos LLC, Ann Arbor, MI, USA), and the values obtained were used to correct urinary BCE concentrations. Measurements of serum CTx was performed using a sandwich immunoassay with two monoclonal antibodies specific for the beta-isomerized 8-amino acid sequence of the C-terminal telopeptide of type I collagen. A calibrator, control, or unknown serum sample is incubated with biotinylated antibody for 9 minutes. After the addition of ruthenium-labeled antibody and streptavidin-coated paramagnetic microbeads, a sandwich complex is formed and binds to the bead via a biotin-streptavidin interaction. After an additional 9 minute incubation, the reaction mixture is aspirated into the measuring cell, where the electrochemiluminescent signal is generated by the ruthenium-labels sandwich complex. All of the above steps were performed automatically by the Elecsys 2010 analyzer.

### 2.6. Statistical Methods

Sample size estimation was performed by precision analysis, using intraclass correlation coefficients (ICCs) [19]. The ICC target value was 0.8 and the 95% confidence interval width was set at 0.2 for the three repeated measurements. Using these values, a sample size of 36 was required [20]. Data normality was tested using the Kolmogorov-Smirnov test. Precision for repeated measurements was performed using ICCs and 95% confidence intervals assuming a two-way random effect model, single measurement, and absolute agreement. Correlation between the lateral flow based immunoassay and ELISA measurements was analyzed, using Pearson's correlation coefficients. Curve estimation regression analysis was performed to determine the exponential relationship between the lateral flow-based immunoassay and ELISA measurements. Statistical analysis was conducted using SPSS for Windows (SPSS V15.0K, SPSS Inc., Chicago, IL, USA). Null hypotheses of no difference were rejected if p-values were less than 0.05.

## 3. Results and Discussion

Four male and 32 female subjects were finally included. Their mean age was 66.2 years (SD 7.5 years) (Table 1).

The precision of the newly developed lateral flow based immunoassay were 0.974 (95% CI, 0.955 to 0.986) for urinary NTx and 0.995 (95% CI, 0.991 to 0.997) for serum CTx. The correlation between the lateral flow based immunoassay was 0.913 (*p* < 0.001) for urinary NTx and 0.872 (*p* < 0.001) for serum CTx. In linear regression analysis the following equations were derived (X relative optical density read by Image J):

Urinary (Figure 6):

$$\text{NTx}\;=\;3955.83\times {\text{e}}^{-4.59\times \text{X}}\;\left({\text{R}}^{2}=0.925\right)$$

Serum (Figure 7):

$$\text{CTx}\;=\;49098440.16\times {\text{e}}^{-0.4819\times \text{X}}\;\left({\text{R}}^{2}=0.688\right)$$

A newly developed lateral flow-based immunoassay measuring urinary NTx and serum CTx was found to be clinically feasible. Repeated measurements showed relevant precision and correlation with the ELISA test. The lateral flow-based immunoassay compared well with the ELISA test in measuring urinary NTx and serum CTx.

Before discussing the study results in detail, some limitations need to be addressed. First, this study contained a somewhat small number of samples, and the most of the subjects were women, perhaps because osteoporosis is far more prevalent in women. The precision of repeated measurements, along with the correlation with ELISA test, needs to be evaluated for a wider range of values with more subjects. Second, in a regression analysis, the relationship between the optical density of lateral flow-based immunoassay and ELISA test was assumed to be exponential. However, the relationship between optical density changes and material concentrations was estimated within a relatively small range of concentrations. Further investigation is needed with a wider range of concentration of the analytes to more broadly validate the test.

Of the bone turnover markers, CTx is a crosslink peptide sequence of type I collagen, which is the most important organic constituent of the bone matrix. This peptide sequence relates to bone turnover because it is the portion that is cleaved by osteoclasts during bone resorption, and its serum levels are proportional to osteoclastic activity [21]. It is also known to be more specific to bone resorption than other markers [11]. NTx is another bone resorption marker derived from type I collagen. NTx can be measured noninvasively in urine samples, but its level fluctuates widely as much as 50% from day to day [22], which sometimes make the interpretation of NTx changes difficult. However, urinary NTx is reported to have a place in clinical practice for the early identification of non-compliant patients or presence of secondary osteoporosis [23].

Although measurements of serum CTx and urinary NTx using the lateral flow-based immunoassay might not be as accurate as those of ELISA, lateral flow-based immunoassay needs less time and costs less than ELISA. Considering that the bone turnover markers have significant diurnal variations [24], and therefore need to be measured repetitively, lateral flow based immunoassay can be a rewarding method measuring of serum CTx and urinary NTx in clinical practice. Measurement using lateral flow-based immunoassay could be improved by selection of more feasible antigens, manufacturing more refined gold nanoparticles, optimization of the lateral flow by matrix stability, and refined optical systems for detecting optical changes.

## 4. Conclusions

A newly developed lateral flow-based immunoassay measuring urinary NTx and serum CTx showed relevant precision of repetitive measurements and correlation with the standard ELISA tests. It is a clinically feasible and productive method of measuring urinary NTx and serum CTx for osteoporosis.

## Figures and Tables

**Figure 6.**
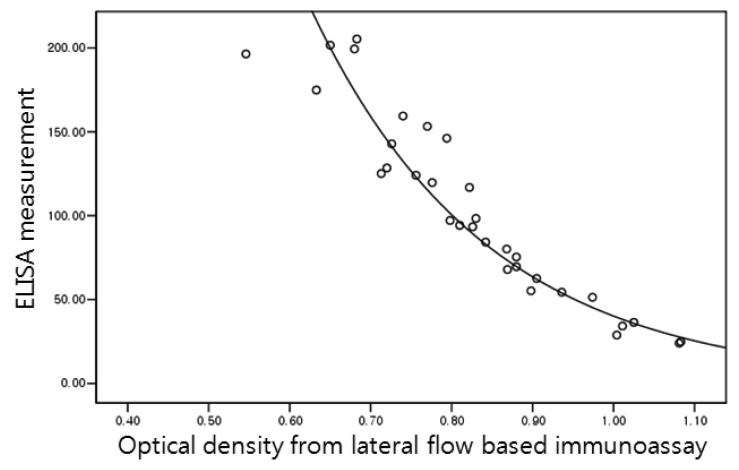
Regression graph of urinary NTx. Transverse axis represents optical density changes from lateral flow based immunoassay and vertical axis ELISA measurement.

**Figure 7.**
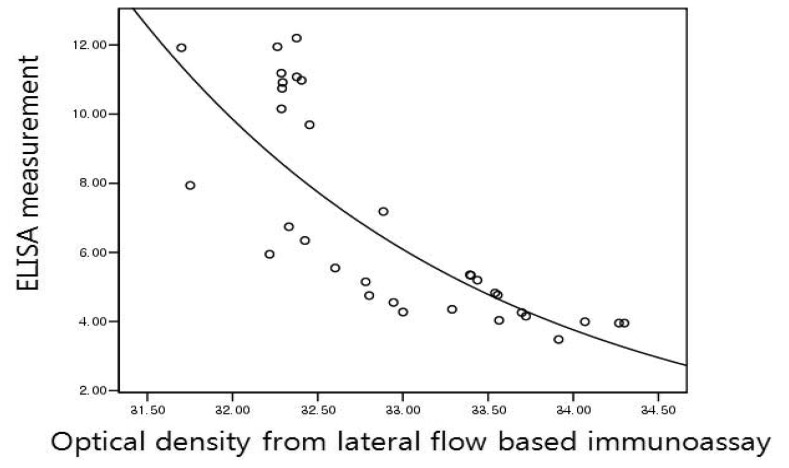
Regression graph of serum CTx. Transverse axis represents optical density changes from lateral flow based immunoassay and vertical axis ELISA measurement.

**Table 1.** Demographic data and summary of measurements.

N	36
Age (years)	66.2 (SD 7.5)
Sex (M:F)	4 : 32
Measurements	
Serum CTx	8.1 (3.1) ng/mL
Urine NTx	121.6 (80.1) nmol BCE/mmol creatinine

Data are presented as mean (SD); BCE: Bone Collagen Equivalence.
